# A pilot cohort study assessing the feasibility of complete revascularization with balloon pulmonary angioplasty for chronic thromboembolic pulmonary hypertension

**DOI:** 10.1371/journal.pone.0254770

**Published:** 2021-07-16

**Authors:** Shinya Fujii, Shinya Nagayoshi, Kazuo Ogawa, Makoto Muto, Toshikazu D. Tanaka, Kosuke Minai, Makoto Kawai, Michihiro Yoshimura

**Affiliations:** 1 Division of Cardiology, Saitama Cardiovascular Respiratory Center, Kumagaya, Saitama, Japan; 2 Division of Cardiology, Department of Internal Medicine, The Jikei University School of Medicine, Tokyo, Japan; Stony Brook University Renaissance School of Medicine, UNITED STATES

## Abstract

Balloon pulmonary angioplasty improves prognosis by alleviating pulmonary hypertension in patients with chronic thromboembolic pulmonary hypertension, even with incomplete revascularization. However, hypoxia or the requirement for pulmonary vasodilators often remain even after pulmonary hypertension relief. With this cohort study, we aimed to examine whether complete revascularization by additional balloon pulmonary angioplasty on residual lesions, even after pulmonary hypertension relief, could resolve hypoxia or the requirement for pulmonary vasodilators. During complete revascularization with balloon pulmonary angioplasty in 42 patients with chronic thromboembolic pulmonary hypertension, we investigated therapeutic effects at baseline (T1), pulmonary hypertension relief phase (T2), and at 6 months post-final balloon pulmonary angioplasty (T3). The pulmonary hypertension relief phase was defined as the first time that a mean pulmonary artery pressure ≤ 25 mmHg or pulmonary vascular resistance ≤ 240 dyn-s/cm^5^ was reached in right heart catheterization before balloon pulmonary angioplasty. The partial pressure of oxygen increased progressively over T1, T2, and T3 (59.2±8.5, 69.0±9.7, and 80.0±9.5 mmHg, respectively; P<0.001 T2 vs. T3). Minimum oxygen saturation levels during the 6-minute walk distance test were 87% (81‒89%), 88% (84‒92%), and 91% (89‒93.3%), respectively (P<0.001 T2 vs. T3), with gradual increase in the 6-minute walk distance (346±125 m, 404±90 m, 454±101 m, respectively; P<0.001 T2 vs. T3). The percentages of patients using pulmonary vasodilators (54.8%, 45.2%, 4.8%, respectively; P<0.001 T2 vs. T3) and requiring oxygen therapy (26%, 26%, 7%, respectively; P = 0.008 T2 vs. T3) decreased significantly without hemodynamic exacerbation or major complications. Despite the discontinuation of pulmonary vasodilators, mean pulmonary artery pressure improved (36.0 [31.0‒41.3], 21.4±4.2, 18.5±3.6 mmHg, respectively; P<0.001 T2 vs. T3). Complete revascularization with balloon pulmonary angioplasty beyond pulmonary hypertension relief benefits patients with chronic thromboembolic pulmonary hypertension; it may improve oxygenation and exercise capacity, and reduce the need for pulmonary vasodilators and oxygen therapy.

## Introduction

Chronic thromboembolic pulmonary hypertension (CTEPH) is a progressive pulmonary vasculature disease caused by organized chronic thrombi and fibrous tissue [1, 2]. Pulmonary endarterectomy (PEA) is a curative therapy and is the gold standard treatment for CTEPH. However, it is not possible to perform PEA in all patients with CTEPH. Residual pulmonary hypertension (PH) after PEA occurs in 17‒51% of cases [3–7]. Re-operative PEA is feasible in these patients, but in-hospital mortality is higher with re-operative PEA [8].

In contrast, balloon pulmonary angioplasty (BPA) is a treatment option for patients with inoperable CTEPH [9–11], those with an unacceptable surgical risk-benefit ratio but who are technically operable, and those with residual PH after PEA [12–14]. The contemporary treatment strategy for CTEPH is a complementary combination of PEA, BPA, and medical therapy.

An important therapeutic goal of BPA is the relief of PH, and previous studies have shown that BPA improves PH [9–11, 15, 16]. BPA aims to achieve a mean pulmonary arterial pressure (mPAP) ≤ 30 mmHg, preferably ≤ 25 mmHg, on right heart catheterization (RHC). Additionally, patients with CTEPH with improvement in PH after BPA therapy have an improved long-term prognosis [17]. However, in approximately half of the patients with CTEPH who undergo BPA therapy pulmonary vasodilators and oxygen therapy are still required [17], and over half of the patients with CTEPH who undergo BPA therapy remain in the World Health Organization (WHO) functional class II [18]. These aspects represent the next challenge in CTEPH treatment.

We hypothesized that complete revascularization with BPA may contribute to improved hypoxia or a reduced need for pulmonary vasodilators and oxygen therapy in patients with CTEPH, and sought to investigate this hypothesis with this cohort study.

## Materials and methods

### Participants

We enrolled 45 consecutive patients with CTEPH who underwent BPA between June 2014 and April 2020 at the Saitama Cardiovascular Respiratory Center and the Jikei University Hospital. We excluded patients whose exercise function could not be assessed due to diseases of the musculoskeletal and nervous system, and included them following PEA. These patients were diagnosed with CTEPH based on standard criteria [1, 19]. All patients were diagnosed as inoperable by an experienced cardiovascular surgeon based on lesion accessibility, hemodynamics, age, and comorbidities. The main reasons for inoperability (from most to least common) were distal lesions, age, patient preference, and comorbidities.

### Balloon pulmonary angioplasty procedure

A series of BPA procedures, including pulmonary artery catherization, lesion wire penetration, and balloon dilation, were performed through the femoral vein. The pulmonary artery was selected by inserting a 6- or 7-French long sheath into the main stem of the pulmonary artery and then inserting a 6- or 7-French guiding catheter (Multipurpose Profit, Judkins right 4.0 Profit, Judkins left 4.0 Profit, and Amplatz Left 1.0 Profit; Nipro Co, Osaka, Japan) into the sheath. If more distal lesions were to be approached selectively or if additional force was needed for a lesion, an additional 6- or 7-French guiding extension catheter (Guideliner PV V3; JAPAN LIFELINE Co, Tokyo, Japan, GUIDEZILLA II PV; Boston Scientific Co, Boston, USA) was inserted into the guiding catheter.

We used a 0.035-inch wire (Radifocus; TERUMO Co., Tokyo, Japan) when selecting pulmonary arteries with guiding catheters; we also used a 0.035-inch wire to pass through the lesion and to maintain the coaxiality of the guiding catheter with the target lesion.

Target lesions were passed through using a 0.014-inch soft wire (Bpahm; JAPAN LIFELINE Co., Tokyo, Japan or Jupiter FC; Boston Scientific Co., Boston, USA) as the first choice, with tip loads ranging from 0.6 g to 1.0 g, and a monorail-type balloon was used for dilatation.

As an anticoagulant during the procedure, 1000‒2000 units of unfractionated heparin were initially administered intravenously, and 500‒1000 units of additional unfractionated heparin were administered intravenously every hour thereafter. During the procedure, oxygen was administered to all patients at a flow rate of 5‒10 L/min.

We dilated as many lesions as possible in a single session [20], with a maximum radiographic time of 90 minutes, a maximum exposure of 2000 mGy, and used up to 300 ml of iodine contrast media. The priority of lesions was as follows: right lung > left lung, lower lobe > middle lobe > upper lobe, type A (ring-like stenosis) > type B (web or band lesion) > type C (subtotal occlusion) > type D (chronic total occlusion) > type E (tortuous lesion) [10, 21, 22], and proximal lesion > distal lesion.

### Treatment strategies and balloon size determination

The therapeutic goal of BPA was to reach a mPAP ≤ 25 mmHg or pulmonary vascular resistance (PVR) ≤ 240 dyn-s/cm^5^. We adjusted the balloon size to avoid vascular injury and aggravation due to excessive dilatation; a balloon with a diameter smaller than that of the vessel was chosen when mPAP exceeded 30 mmHg before BPA. Consequently, owing to the limitation of the balloon size, the lesion may not be sufficiently dilated. We hypothesized that the treatment of residual lesions may contribute to improving the quality of life (QOL) of patients with CTEPH, which is the next therapeutic goal of BPA. Therefore, our strategy was to treat any residual lesions on pulmonary angiography aggressively, even in patients with mPAP ≤ 25 mmHg or PVR ≤ 240 dyn-s/cm^5^. We terminated all BPA once there were no treatable residual lesions on pulmonary angiography verified with perfusion scintigraphy.

**Fig 1:**
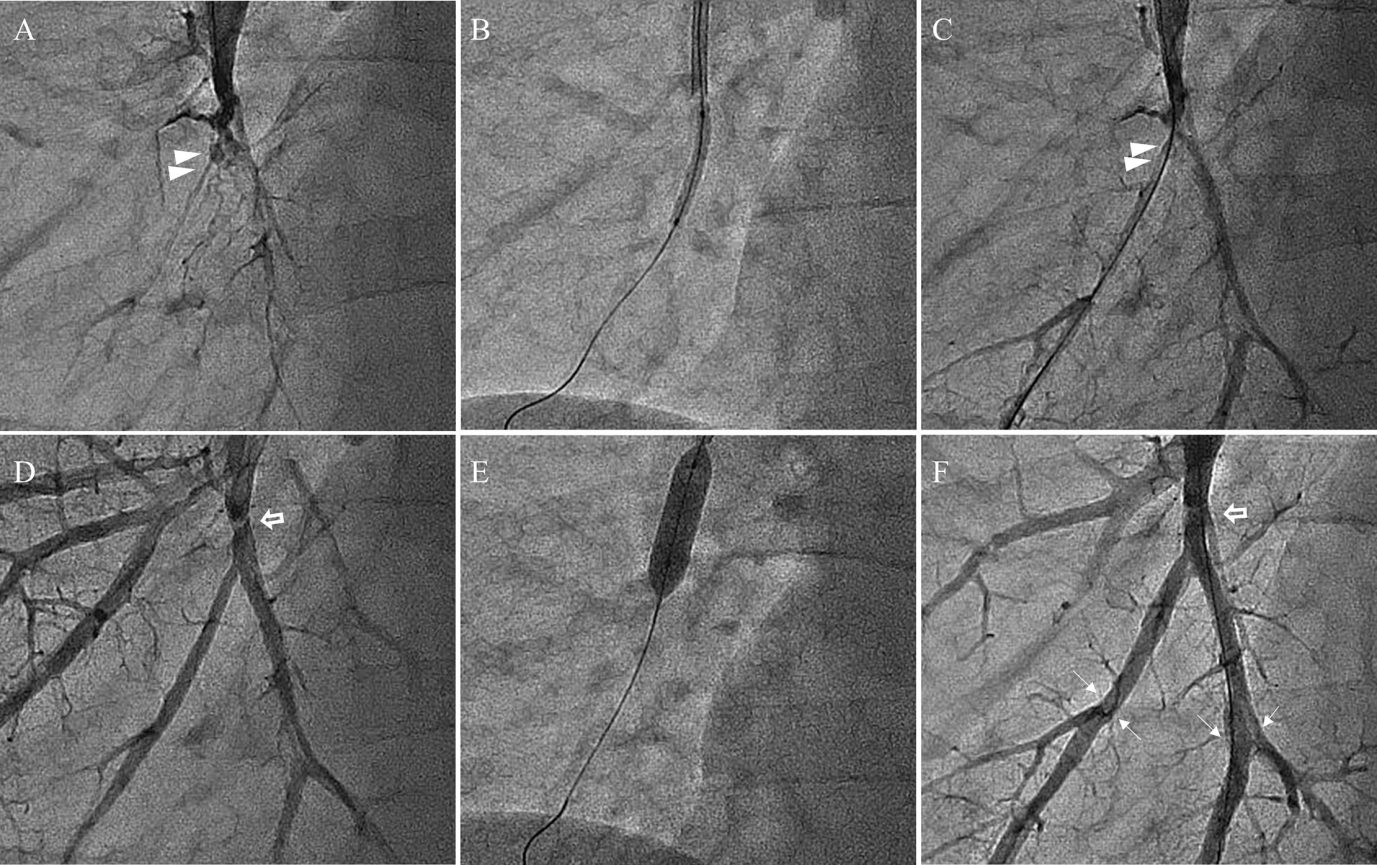
Balloon pulmonary angioplasty of the right pulmonary basilar artery (A10). (A) Pulmonary angiography before the first balloon pulmonary angioplasty (BPA). Subtotal occlusion (arrowhead) of the right A10. (B) The 2.0-mm balloon is dilated at 6 atmospheres in the right A10. (C) Pulmonary angiography immediately after the first BPA of the right A10 (arrowhead) shows a dilated vessel and increased flow in the distal artery. (D) Pulmonary angiography of the right A10 before BPA during the third session showing reduced pulmonary hypertension. The banded lesion (arrow) remains on the right A10. (E) The balloon size is determined visually, and the 5.5-mm balloon is dilated to 4 atmospheres. (F) Pulmonary angiography of the third BPA after overdilation of the balloon in the proximal part of the right A10 (arrow) and after dilatation of the distal bifurcation lesion (thin arrow). Extravasation of contrast media was not noted when the balloon was overdilated, and residual banded lesions can be observed (arrow). If pulmonary hypertension improved, then no major vascular injury occurred when the balloon was overdilated.

Moreover, if mPAP before BPA was ≤ 30 mmHg, a selection of a balloon with a diameter exceeding the vessel diameter was considered acceptable for residual lesions while attempting to achieve a mPAP ≤ 25 mmHg or PVR ≤ 240 dyn-s/cm^5^ (Fig 1). If mPAP was ≤ 30 mmHg before the initial BPA, no sizing was performed and a balloon size greater than the vessel diameter was allowed for lesion dilation. If resistance was felt when advancing the wire in the distal vessel, the vessel was treated as far distally as possible because of the presence of a chronic thrombus. However, treatment of the distal area was performed after PH relief whenever possible. If the vessel diameter of the distal lesion was ≤ 2.0 mm, the patient was treated with either a 2.0-mm balloon with low-pressure dilation or with only balloon passage, without dilation.

### Complication and countermeasures

It is considered that most pulmonary injuries after BPA are due to mechanical vascular injury caused by the perforation of the pulmonary artery by the wire or balloon overdilation [23, 24]. Therefore, in the present study, when wet cough, bloody sputum, and associated hypoxia persisted, hemostatic procedures, such as a wedging of guiding catheters, hemostasis by blocking blood flow with balloon dilation, and injection of a gelatin sponge, were aggressively performed on the target vessels.

All patients underwent chest radiography and chest computed tomography (CT) within 1 hour after BPA. The evaluation categories for complications were hemostasis, blood sputum, decreased oxygen saturation (SpO_2_) (>5%) during or after the procedure under oxygen administration, opacity on chest radiograph and chest CT, requirement for noninvasive positive pressure ventilation, intratracheal intubation, requirement for veno-arterial extracorporeal membrane oxygenation (VA-ECMO), and death.

The categories were counted based on the presence of the complication to determine its incidence. If more than one category was present, all categories were counted as positive.

### Study design

A baseline assessment was performed prior to starting BPA. Thereafter, BPA was performed repeatedly, and all BPA therapies were terminated based on the operator’s subjective judgment on whether there were any residual lesions requiring treatment by the end of the session. Subsequent BPA sessions were conducted for at least one week but less than two months following the previous session. The effects of a single BPA cannot be accurately assessed immediately after BPA due to the influence of contrast media and lung damage. Therefore, the therapeutic effect of each BPA was assessed immediately before the following session. The therapeutic effect of the final session was measured approximately 6 months after the session and included RHC assessments.

In this cohort, the treatment effects of BPA were evaluated at the following three time-points:

Baseline (T1): At RHC before the first BPA was evaluated.
The PH relief phase (T2): In the process of repeating BPA, we defined the PH relief phase as the time-point when a mPAP ≤ 25 mmHg or PVR ≤ 240 dyn-s/cm^5^ was reached for the first time during RHC before the beginning of each subsequent BPA session. The PH relief phase was designed to irrespective of pulmonary vasodilator use.
Six months post-final BPA session (T3): At RHC 6 months after the final BPA session.

The primary endpoints included WHO functional classification, hemodynamics on RHC, oxygenation by blood gas analysis, blood collection data such as hemoglobin, estimated glomerular filtration rate (eGFR) and BNP values, 6-minute walk distance (6MWD), percentage of patients requiring pulmonary vasodilators, and percentage of patients requiring oxygen therapy. RHC, blood gas analysis, and 6MWD test were performed without oxygen supplementation.

Additionally, the procedures and complications in all the BPA sessions were evaluated as secondary endpoints. Furthermore, to assess the risk of BPA performed after the PH relief phase (mPAP ≤ 25 mmHg or PVR ≤ 240 dyn-s/cm^5^), we compared the procedures and complications in BPA sessions between those performed before and after a mPAP ≤ 25 mmHg or PVR ≤ 240 dyn-s/cm^5^ was reached.

### Criteria for discontinuation of pulmonary vasodilators and oxygen therapy

In the present study, pulmonary vasodilators were used in principle if mPAP was above 40 mmHg. At the end of each BPA session, pulmonary vasodilators were discontinued when it was determined that a mPAP ≤ 25 mmHg or PVR ≤ 240 dyn-s/cm^5^ could be reached without pulmonary vasodilators. However, if mPAP ≤ 25 mmHg or PVR ≤ 240 dyn-s/cm^5^ was not reached at RHC before the start of subsequent BPA sessions, pulmonary vasodilators were resumed. If mPAP ≤ 25 mmHg or PVR ≤ 240 dyn-s/cm^5^ was reached without the pulmonary vasodilators, pulmonary vasodilators were not reintroduced. Oxygen therapy was discontinued based on a minimum SpO_2_ ≥ 90% or if the patient remained asymptomatic for ≥ 85% of the 6MWD test.

### Ethical approval

This study was approved by the Ethics Committee of the Saitama Cardiovascular and Respiratory Center and The Jikei University School of Medicine (approval numbers 2019056 and 32–132[10208], respectively) and was conducted in accordance with the Declaration of Helsinki. Written informed consent was obtained from all patients.

### Statistical analyses

Continuous variables are expressed as mean±standard deviation or median (interquartile range), and categorical variables are expressed as numbers and proportions (%). In the analysis of BPA procedure results and complications, comparisons between the two groups were examined using the Mann–Whitney U- and Student’s *t-*tests for continuous variables and the Pearson’s chi-square test or Fisher’s exact test for categorical variables. A P-value <0.05 was considered statistically significant.

Hemodynamic parameters from RHC, 6MWD test data, blood gas analysis data, and blood collection data between the three phases were examined by repeated-measures analysis of variance (Bonferroni’s test with post-hoc analysis) and Friedman’s test (Wilcoxon’s signed-rank test between the two groups with post hoc analysis). In the three-phase analysis of pulmonary vasodilators and oxygen administration, categorical variables were examined using the McNemar test. All analyses were performed using the IBM SPSS Statistics 25 software (IBM, Armonk, NY, USA).

## Results

**Fig 2:**
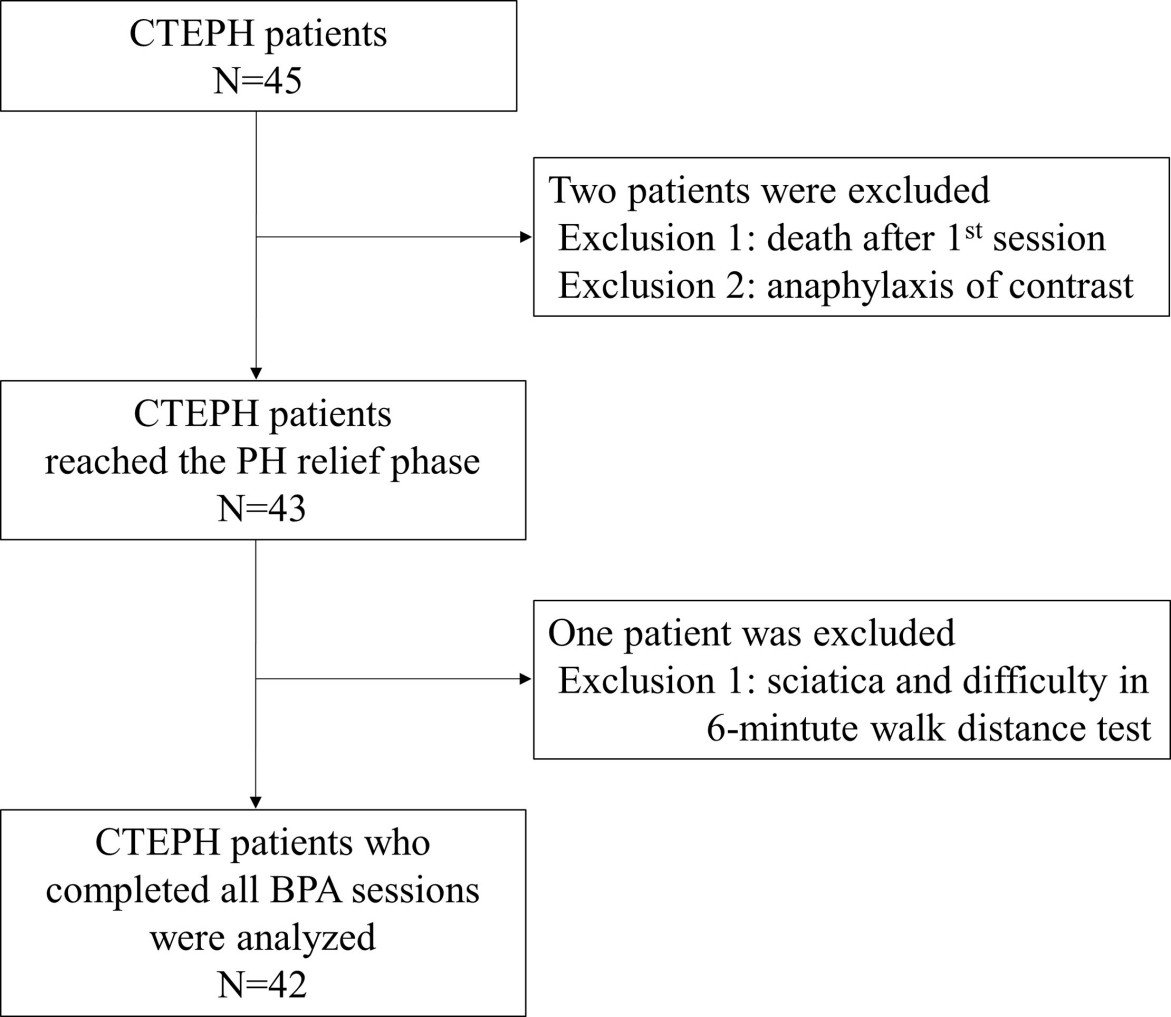
Study flow chart. BPA, balloon pulmonary angioplasty; CTEPH, chronic thromboembolic pulmonary hypertension; PH, pulmonary hypertension. The PH relief phase: The first time a mean pulmonary artery pressure ≤ 25 mmHg or pulmonary vascular resistance ≤ 240 dyn-s/cm^5^ was reached before each subsequent BPA session.

A total of 157 BPA sessions were performed on 45 CTEPH patients (Fig 2), of whom one died within 30 days of the initial BPA from right heart failure associated with multiple lung injuries. One patient still had PH at the second BPA session, but BPA treatment was interrupted due to anaphylactic shock caused by contrast media. In the 63 BPA sessions before the PH relief phase, these two patients withdrew from the study. Forty-three patients reached the PH relief phase within 1‒4 BPA sessions.

Ninety-four BPA sessions were then added for these 43 patients, with at least one additional BPA session per patient. One patient who reached the PH relief phase after three sessions was excluded from the analysis because she was unable to walk due to sciatica after the 4^th^ BPA session and did not perform a 6MWD test. Ultimately, 42 patients completed a total number of 2‒5 BPA sessions (median 3.00, interquartile range 3.00‒4.00 times/person) and were included in the analysis. The therapeutic effect of the final session was measured 196 (180‒361) days after the final BPA was implemented.

The baseline characteristics of the 42 patients included in the analysis are shown in Table 1. The age at first admission ranged from 30 to 84 years. Eighteen patients (42.9%) were classified as WHO functional class III or IV. MPAP was above 40 mmHg in 14 patients (33.3%).

**Table 1:** Patient characteristics at baseline.

	N = 42
Age (years)	69.5 (61.5‒75.8)
Sex (female), n (%)	30 (71.4)
WHO functional class II/III/IV, n (%)	24/17/1 (57.1/40.5/2.4)
Post-pulmonary endarterectomy, n (%)	1 (2.4)
Hb level, g/Dl	13.0 ± 1.8
BNP level, pg/dL	46.3 (18.5‒162.8)
eGFR, mL/min per 1.73 m^2^	57.3 (50.9‒66.5)
Respiratory function and exercise capacity	
VC, L	2.54 (1.83‒2.90)
%VC, %	81 (66.5‒96.3)
FEV1.0, L	1.76 (1.36‒2.21)
FEV1.0% Gaensler, %	75.7 (71.3‒82.8)
PaO_2_ on room air, mmHg	59.2 ± 8.5
Minimum SpO_2_ during 6MWD, %	87 (81‒89)
6MWD, m	346 ± 125
Hemodynamics on right heart catheterization Mean RAP, mmHg	5.0 (3.0‒7.0)
Systolic PAP, mmHg	64 (54.0‒73.0)
Diastolic PAP, mmHg	19 (16.0‒23.3)
Mean PAP, mmHg	36.0 (31.0‒41.3)
PAWP, mmHg	8.7 ± 2.8
Systolic/Diastolic NIBP, mmHg	129.5 (123.3‒144.3)/76 (68.5‒82.8)
HR, beats/min	71 ± 11
SvO_2_	61.0 ± 8.7
CO (thermodilution), L/min	5.0 ± 1.2
CI (thermodilution), L/min per m^2^	3.35 (2.73‒3.64)
SV (thermodilution), ml	71.8 ± 19.8
PVR (thermodilution), dyn-s/cm^5^	454 (328‒586)
Medication for PH, n (%)	23 (54.8)
ERA, n (%)	9 (21.4)
PDE5i, n (%)	3 (7.1)
Oral PGI2	3 (7.1)
SGCs	16 (38.1)
Anticoagulants, n (%)	42 (100)
Oxygen therapy, n (%)	11 (26.2)

Data are presented as mean ± standard deviation, n (%), or median (interquartile range).

6MWD, 6-minute walk distance; BNP, brain natriuretic peptide; BPA, balloon pulmonary angioplasty; CI, cardiac index; CO, cardiac output; eGFR, estimated glomerular filtration rate; ERA, endothelin receptor antagonist; FEV1.0, forced expiratory volume in 1 second; Hb, hemoglobin; HR, heart rate; hemoglobin; NIBP, non-invasive blood pressure; PAP, pulmonary arterial pressure; PAWP, pulmonary arterial wedge pressure; PDE5i, phosphodiesterase-5 inhibitor; PEA, pulmonary endarterectomy; PGI2, prostaglandin I-2; PH, pulmonary hypertension; PVR, pulmonary vascular resistance; RAP, right atrial pressure; sGCs, soluble guanylate cyclase stimulator; SV, stroke volume; VC, vital capacity; WHO, World Health Organization.

### Balloon pulmonary angioplasty procedure

The procedures and complications of all 157 BPA sessions and a comparison of the 63 BPA sessions performed before the PH relief phase and the 94 BPA sessions performed after a mPAP ≤ 25 mmHg or PVR ≤ 240 dyn-s/cm^5^ was reached, are shown in Table 2. Because we dilated as many lesions as possible in a single session, the number of lobes and vessels dilated in one session was high.

**Table 2:** Comparison of procedural results and complications for each session of balloon pulmonary angioplasty.

	All BPA	BPA before the PH relief phase*	BPA after the PH relief phase	P-value
	N = 157	N = 63	N = 94	
Procedural results				
No. of treated segments/procedure	7 (6‒9)	8 (6‒9)	7 (5‒8)	**0.006**
No. of treated vessels/procedure	16.1 ± 6.5	16.4 ± 6.5	15.9 ± 6.5	0.635
Treatment of Type D lesion, n (%)	32 (20.5)	10 (16.1)	22 (23.4)	0.251
Treatment of Type E lesion, n (%)	2 (1.3)	0	2 (2.1)	0.357
Fluoroscopy time/procedure, min	97 (83‒114)	91 (79‒109)	102 ± 21	**0.042**
Amount of contrast medium/procedure, mL	260 (210‒280)	234 ± 67	270 (231‒280)	**0.022**
Radiation dose, mGy	1047 (680‒1641)	993 (655‒1639)	1056 (713‒1614)	0.553
Hemostat procedure				
Balloon occlusion or catheter wedge, n (%)	30 (19.1)	12 (19.0)	18 (19.1)	0.987
Gelatin sponge injection, n (%)	20 (12.7)	10 (15.9)	10 (10.6)	0.335
Any hemostat procedure, n (%)	43 (27.4)	18 (28.6)	25 (26.6)	0.786
Mild-moderate complication				
Opacity on chest radiograph, n (%)	19 (12.1)	8 (12.7)	11 (11.7)	0.851
Opacity on chest CT, n (%)	101 (64.3)	44 (69.8)	57 (60.6)	0.238
Bloody sputum, n (%)	28 (17.8)	11 (17.5)	17 (18.1)	0.92
Decrease of SpO_2_ >5%, n (%)	9 (5.7)	3 (4.8)	6 (6.4)	0.741
Allergy to contrast media, n (%)	4 (2.5)	3 (4.8)	1 (1.1)	0.178
Severe complication				
NPPV, n (%)	5 (3.2)	4 (6.3)	1 (1.1)	0.085
Intratracheal intubation, n (%)	2 (1.3)	2 (3.2)	0	0.159
VA-ECMO, n (%)	1 (0.6)	1 (1.6)	0	0.401
Death, n (%)	1 (0.6)	1 (1.6)	0	0.401

Data are presented as mean ± standard deviation, n (%), or median (interquartile range). BPA, balloon pulmonary angioplasty; CT, computed tomography; NPPV, non-invasive positive pressure ventilation; PH, pulmonary hypertension; VA-ECMO; veno-arterial extracorporeal membrane oxygenation; SpO_2_, oxygen saturation; no., number.

*The PH relief phase: In the process of repeated BPA, the time when a mean pulmonary artery pressure ≤ 25 mmHg or pulmonary vascular resistance ≤ 240 dyn-s/cm^5^ was reached for the first time in right heart catheterization before the beginning of each subsequent BPA session.

### Complications of balloon pulmonary angioplasty

The incidence of opacity on chest CT was significantly higher than the frequency of hemostatic procedures. The pulmonary injury suggested by opacity on the chest CT seemed to include many asymptomatic pulmonary injuries. This was similar between the BPA sessions performed before and after a mPAP ≤ 25 mmHg or PVR ≤ 240 dyn-s/cm^5^ was reached.

Two patients required intratracheal intubation; one for anaphylactic shock by contrast media and the other for pulmonary injury. The second patient, classified as WHO functional class IV with a mPAP of 46 mmHg, underwent VA-ECMO and died within 30 days of the initial BPA.

There were no significant differences between the BPA sessions performed before and after a mPAP ≤ 25 mmHg or PVR ≤ 240 dyn-s/cm^5^ was reached in the incidence of asymptomatic to moderate lung injury events, such as opacity on chest radiograph and CT, bloody sputum, and decreased SpO_2_. Moreover, intratracheal intubation, VA-ECMO placement, and death, as advanced pulmonary injury events, were not observed in the sessions after a mPAP ≤ 25 mmHg or PVR ≤ 240 dyn-s/cm^5^ was reached. One case of noninvasive positive pressure ventilation placement in the sessions after a mPAP ≤ 25 mmHg or PVR ≤ 240 dyn-s/cm^5^ was reached involved wire perforation into the extravascular region and balloon dilation in the treatment of a type D lesion.

### Changes in hemodynamic parameters on right heart catheterization

**Fig 3:**
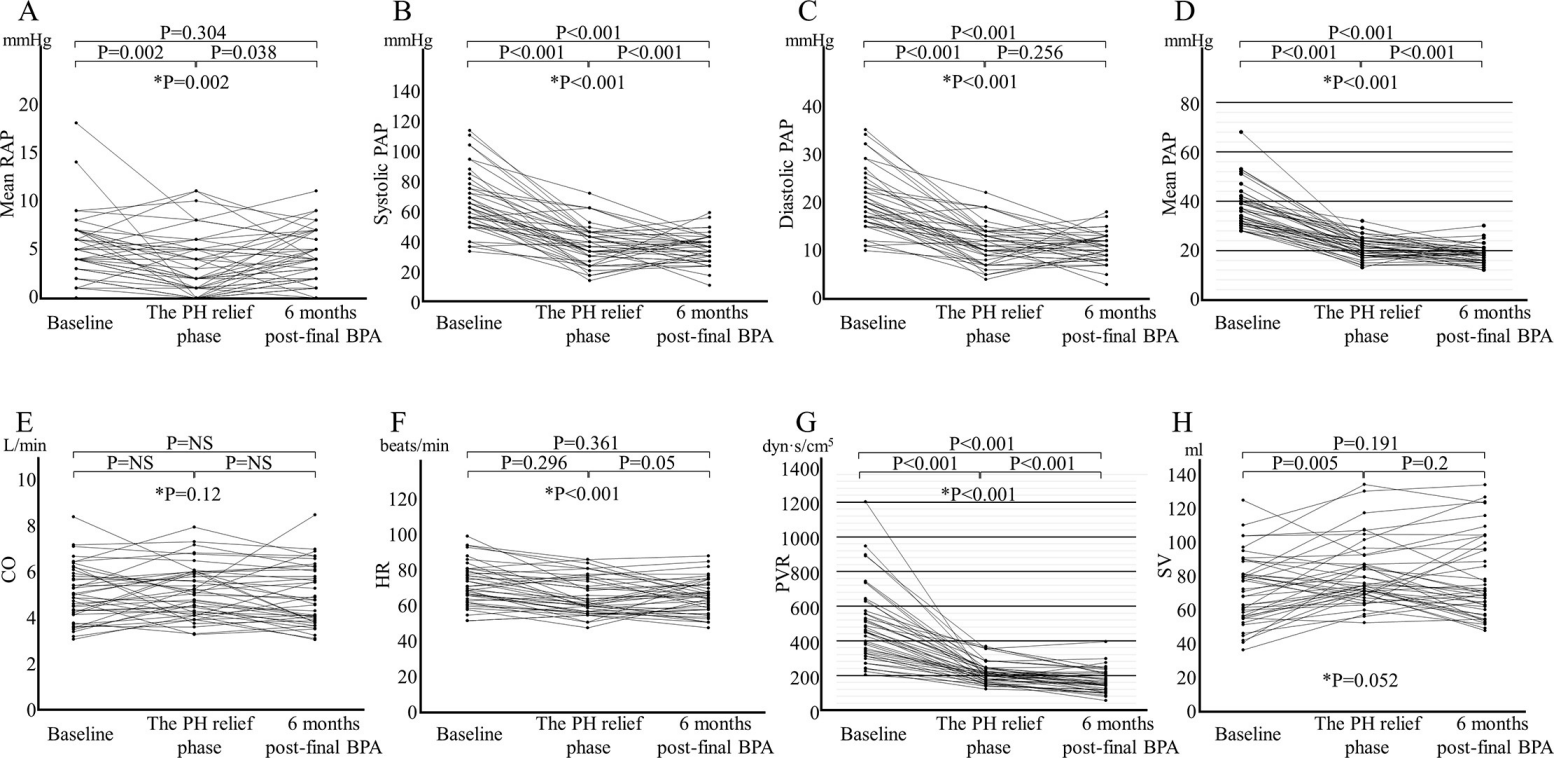
Changes in the parameters of right heart catheterization. (A) Mean right atrial pressure (RAP). (B) Systolic pulmonary artery pressure (PAP). (C) Diastolic PAP. (D) Mean PAP. (E) Cardiac output (CO). (F) Heart rate (HR). (G) Pulmonary vascular resistance (PVR). (H) Stroke volume (SV). In the graph showing brain natriuretic peptide (BNP) and estimated glomerular filtration rate (eGFR), the case represented by the dotted line is a patient on dialysis, with higher BNP level transitions and lower eGFR levels at all time points. The pulmonary hypertension (PH) relief phase was the first-time a mean PAP ≤ 25 mmHg or PVR ≤ 240 dyn-s/cm^5^ was reached before each subsequent balloon pulmonary angioplasty (BPA) session. *P-value of Friedman’s test.

Fig 3 shows the changes in the hemodynamic parameters of RHC at the three-time points. MPAP and PVR in the PH relief phase were 21.4±4.2 mmHg and 208 (171‒243) dyn-s/cm^5^, respectively. These values were sufficient for the conventional treatment goal of BPA. Additional BPA was performed until no treatable residual lesions remained. MPAP and PVR after the final BPA were further reduced to 18.5±3.6 mmHg (P = 0.043 for T2 vs. T3) and 171±65 dyn-s/cm^5^ (P<0.001 for T2 vs. T3), respectively. Between T2 and T3, systolic PAP significantly improved (37.5±8.4 mmHg and 30.9±6.1 mmHg; P = 0.02), and these improvements in a series of hemodynamic parameters were observed despite the withdrawal of pulmonary vasodilators (Table 3).

**Table 3:** Pulmonary vasodilators and oxygen therapy at baseline, at the pulmonary hypertension relief phase, and at 6 months post-final balloon pulmonary angioplasty.

	Baseline	The PH relief phase*	Six months post-final BPA session
	N = 42	N = 42	N = 42
Medication for PH, n (%)	23 (54.8)	19 (45.2)	2 (4.8)†‡
ERA, n (%)	9 (21.4)	5 (11.9)	0 †
PDE5i, n (%)	3 (7.1)	3 (7.1)	0
Oral PGI_2_, n (%)	3 (7.1)	0	0
sGCs, n (%)	16 (38.1)	15 (35.7)	2 (4.8)†‡
Oxygen therapy, n (%)	11 (26.2)	11 (26.2)	3 (7.1)†‡

Categorical variables are indicated by numbers and ratios (%).

BPA, balloon pulmonary angioplasty; ERA, endothelin receptor antagonist; PDE5i, phosphodiesterase-5 inhibitor; PGI2, prostaglandin I-2; PH, pulmonary hypertension; sGCs, soluble guanylate cyclase stimulator; vs., versus.

*The PH relief phase: In the process of repeated BPA, the time when a mean pulmonary artery pressure ≤ 25 mmHg or pulmonary vascular resistance ≤ 240 dyn-s/cm^5^ was reached for the first time in right heart catheterization before the beginning of each subsequent BPA session.

†P-value<0.05 (vs. baseline).

‡P-value<0.05 (vs. the PH relief phase).

Diastolic PAP did not change significantly (T2, 11.4±4.0 mmHg; T3, 10.5±3.0 mmHg; P = 0.256). Cardiac output varied among the three phases and did not change significantly. There was a decreasing trend in heart rate over time: T1, 71±12 bpm; T2, 61 bpm (56‒74 bpm); and T3, 64±10 bpm. Stroke volume showed an increasing trend (T1, 71.8±19.8 mL; T2, 74.1 mL [68.8‒88.0]; and T3, 69.9 mL [59.3‒96.4]) but did not change consistently, and the changes were not significant.

In the present study, all 42 patients ultimately reached a mPAP ≤ 25 mmHg or PVR ≤ 240 dyn-s/cm^5^ at T3. Forty of the 42 patients (95.2%) reached a mPAP ≤ 25 mmHg at T3. The remaining two patients had a mPAP > 25 mmHg, but reached PVR ≤240 dyn-s/cm^5^. In addition, 33 of the 42 patients (78.6%) finally reached a mPAP ≤ 20 mmHg at T3. This result was reached despite low pulmonary vasodilator usage (4.8%).

### Changes in exercise capacity, oxygenation, and laboratory data

**Fig 4:**
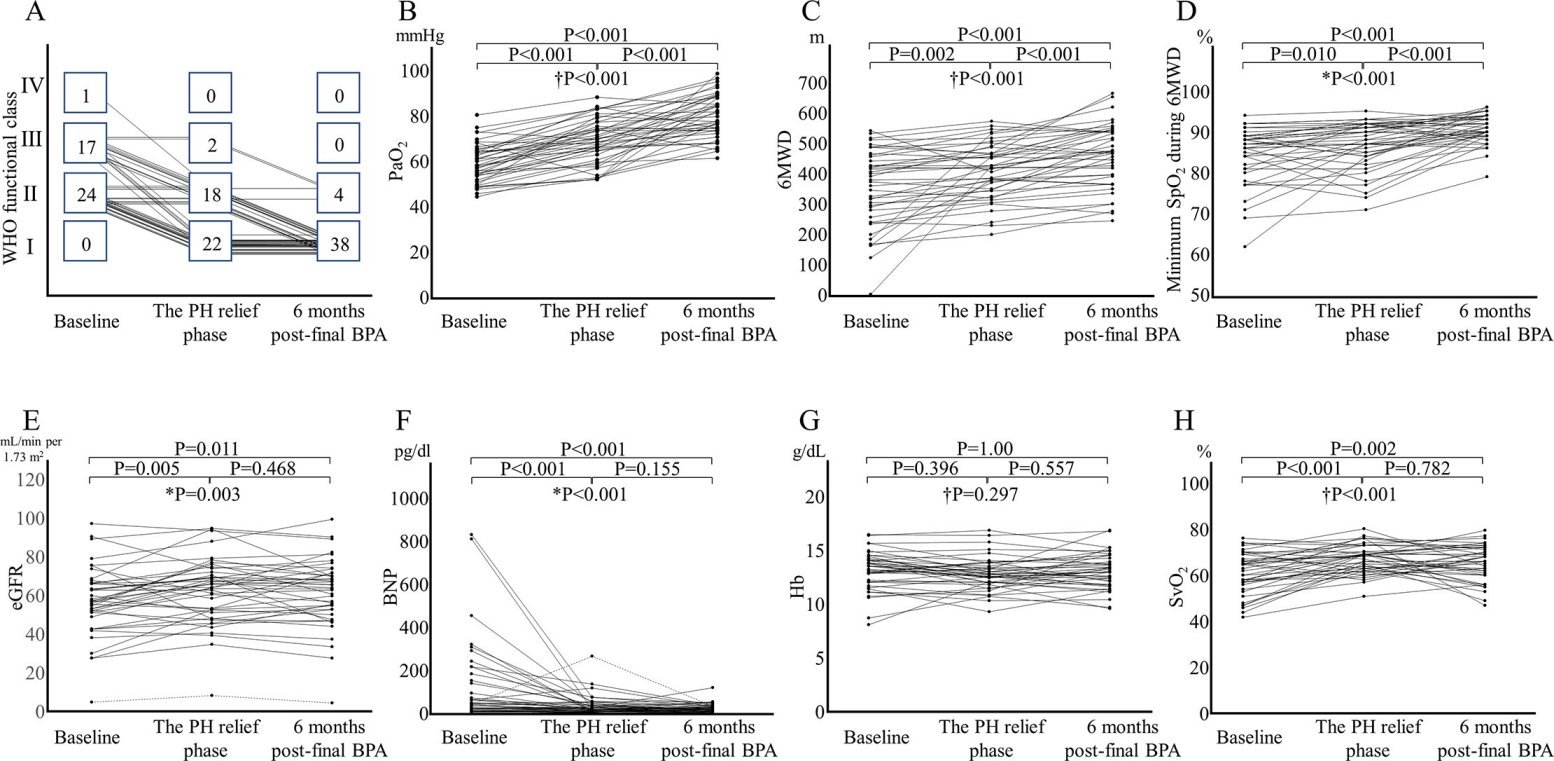
Changes in oxygenation, exercise capacity, and laboratory data. (A) World Health Organization (WHO) functional class. (B) The partial pressure of oxygen (PaO_2_). (C) The 6-minute walk distance (6MWD) test. (D) Minimum oxygen saturation (SpO_2_) during the 6MWD test. (E) Estimated glomerular filtration rate (eGFR). (F) Brain natriuretic peptide (BNP). (G) Hemoglobin (Hb) level. (H) Mixed venous oxygen saturation (SvO_2_). The pulmonary hypertension (PH) relief phase was the first time a mean pulmonary artery pressure ≤ 25 mmHg or pulmonary vascular resistance ≤ 240 dyn-s/cm^5^ was reached before each subsequent balloon pulmonary angioplasty (BPA) session. *P-value of Friedman’s test. †P-value for repeated-measures analysis of variance.

Fig 4 graphically shows changes in the WHO functional classification, blood gas analysis, BNP, eGFR, Hb, and 6MWD values during the three phases. When the PH relief phase was reached, 2 (4.8%) patients remained in WHO functional class III, and 18 (42.9%) patients remained in WHO functional class II. However, 6 months after the final BPA, no WHO functional class III patients were identified, and the number of WHO functional class II patients had improved to 4 (9.5%).

Furthermore, in arterial blood gas analysis, the partial pressure of oxygen, which is an indicator of oxygenation at rest, consistently improved over time, with statistically significant differences (T1, 59.1±8.4 mmHg; T2, 69.0±9.7 mmHg; T3, 80.0±9.5 mmHg; P<0.001 for all pairwise comparisons). During the 6MWD test, the minimum SpO_2_, which indicates oxygenation during exercise, also improved consistently over time, with statistically significant differences (T1, 87% [81%‒89%]; T2, 88% [84%‒92%]; T3, 91% [89%‒93.3%]; P<0.001 for all pairwise comparisons). The 6MWD values for the minimum SpO_2_ did not decrease; instead, these consistently increased at each time point (T1, 346±127 m; T2, 404±90 m; and T3, 453±101 m; P = 0.002 for T1 vs. T2; P<0.001 for T2 vs. T3 and T1 vs. T3). These improvements in oxygenation and exercise capacity were observed despite the withdrawal of pulmonary vasodilators (Table 3).

The BNP levels decreased between T1 and T2 (46.3 pd/dL [18.5‒162.8] and 21.8 pg/dL [14.5‒43.6], respectively; P<0.001) and did not change significantly between T2 and T3 (21.8 pg/dL [14.5‒43.6] and 23.4 pg/dL [12.2‒36.6], respectively; P = 0.155). eGFR and mixed venous oxygen saturation (SvO_2_) improved with BPA therapy but did not improve from T2 to T3 (eGFR: T1, 57.3 mL/min/1.73 m^2^ [50.9‒66.5]; T2, 64.3 mL/min/1.73 m^2^ [50.6‒72.6]; T3, 63.8 mL/min/1.73 m^2^ [52.4‒71.7]; P = 0.005 for T1 vs. T2; P = 0.468 for T2 vs. T3; P = 0.011 for T1 vs. T3; SvO_2_: T1, 61.0%±8.7%; T2, 67.1%±6.1%; T3, 65.6%±7.7%; P<0.001 for T1 vs. T2; P = 0.782 for T2 vs. T3; P = 0.002 for T1 vs. T3). Hb slightly changed during BPA therapy (T1, 13.0±1.8; T2, 12.7±1.5; T3, 13.0±1.6).

### Changes in the need for pulmonary vasodilators and oxygen therapy

Appropriate additional BPA allowed most patients to discontinue the use of pulmonary vasodilators and oxygen therapy (Table 3). In this study, pulmonary vasodilators were actively discontinued based on the subjective judgment that a mPAP ≤ 25 mmHg or PVR ≤ 240 dyn-s/cm^5^ could be reached without pulmonary vasodilators at the end of each BPA session. This decision was guided by mPAP ≤ 25 mmHg or PVR ≤ 240 dyn-s/cm^5^ on the RHC measured at the end of each BPA session. Even if these criteria at the end of each BPA session were not strictly fulfilled, pulmonary vasodilators were actively discontinued if the BPA session was performed extremely well. Pulmonary vasodilators were discontinued within a week after the BPA session. As a result, fewer patients required pulmonary vasodilators at T2 than at T1. Following the T2 time point, pulmonary vasodilators were discontinued when it was determined that subsequent additional BPA sessions had been successfully completed and a mPAP ≤ 25 mmHg or PVR ≤ 240 dyn-s/cm^5^ could be reached without pulmonary vasodilators at the end of each additional BPA session. This decision also was based on the same guidance as described above. As a result, fewer patients required pulmonary vasodilators at T3 than at T2, pulmonary vasodilators at T2 and T3 were required by 19 (45.2%) and 2 (4.8%) patients, respectively (P < 0.001). No patient was reintroduced after discontinuation of pulmonary vasodilators due to failure to reach mPAP ≤ 25 mmHg or PVR ≤ 240 dyn-s/cm^5^ without pulmonary vasodilators.

Discontinuation of soluble guanylate cyclase stimulator (sGCs) was the most frequent in pulmonary vasodilators. SGCs was required at T2 and T3 by 15 (35.7%) and 2 (4.8%) patients, respectively (P = 0.001). Discontinuation of sGCs was not possible, even at the end of the final BPA session, for two patients. Oxygen therapy was required at T2 and T3 by 11 (26.2%) and 3 (7.1%) patients, respectively (P = 0.008).

## Discussion

The present study demonstrated four benefits of complete revascularization by additional BPA for residual lesions even after PH relief had been reached: improved hemodynamics on RHC, increased oxygenation capacity, improved exercise capacity, and the withdrawal of oxygen therapy and pulmonary vasodilators.

### Benefits of oxygen therapy withdrawal

In the early days when BPA procedure was established, details of oxygenation with BPA therapy in CTEPH have not been reported [9–13, 15]. However, improvements in oxygenation with BPA in chronic thromboembolic disease (CTED) and CTEPH have been reported in recent years [25, 26]. The present study showed that additional BPA further improved subjective symptoms, oxygenation, and exercise capacity in CTEPH after PH relief. Finally, 8 of 11 patients (72.7%) were able to discontinue oxygen therapy; for CTEPH patients, oxygen therapy-free living can improve the QOL.

### Benefits of pulmonary vasodilator withdrawal

Complete revascularization with BPA achieved pulmonary vasodilator withdrawal in 21 of 23 patients (86.9%) requiring pulmonary vasodilators. Thus, this study showed that complete revascularization with BPA significantly reduced the need for pulmonary vasodilators in patients with CTEPH. The results of the present study also show a much lower rate of pulmonary vasodilator use after BPA therapy than those of previous reports [17, 18, 26]. Meanwhile, it was also shown that a small number of patients with CTEPH were unable to discontinue pulmonary vasodilators due a failure in normalizing pulmonary circulation even after complete revascularization with BPA. It was suggested that complete revascularization with BPA provides significant improvement, but also has certain limitations in the improvement.

### Factors contributing to the therapeutic effect of balloon pulmonary angioplasty

The therapeutic benefit of performing additional BPA is due to two factors. First, the therapeutic effect on untreated lesions. In the present study, pulmonary perfusion scintigraphy was performed before each BPA session and at T3. Pulmonary perfusion scintigraphy deficiencies were found in all cases, even when the PH relief phase was reached. Therefore, BPA was added for an exploration of untreated lesions. The findings of hypoperfusion on scintigraphy and anatomical information of pulmonary vessels on CT were used as references. Second, the additional therapeutic effect on insufficiently dilated lesions. Insufficiently dilated sites seen on pulmonary angiography were re-dilated by allowing the use of a balloon with a diameter approximately 1.5 times the vessel diameter as shown in Fig 1.

We experienced improvement due to the synergistic effect of these two factors. It is important to recognize the presence of residual lesions as this is a prerequisite to performing appropriate additional BPA.

### Risk assessment of balloon pulmonary angioplasty

Asymptomatic to moderate complications were not significantly different between sessions before and after a mPAP ≤ 25 mmHg or PVR ≤ 240 dyn-s/cm^5^ was reached. In terms of lesion priority, refractory and distal lesions, including type D and E lesions, were the target lesions after a mPAP ≤ 25 mmHg or PVR ≤ 240 dyn-s/cm^5^ was reached. This could explain why the frequency of vascular injury between the two groups was comparable. However, the frequency of severe lung injury was lower in sessions after a mPAP ≤ 25 mmHg or PVR ≤ 240 dyn-s/cm^5^ was reached. The results suggest that if vascular injury occurs, mPAP is sufficiently low to prevent severe lung injury. Additional BPA after achieving a mPAP ≤ 25 mmHg or PVR ≤ 240 dyn-s/cm^5^ was shown to be safe and feasible.

This study presented a clinical dilemma regarding the most appropriate revascularization strategy. The strategy of achieving complete revascularization may increase the total number of sessions. In the present study, many lesions were treated in a single session; therefore, a lower total number of sessions led to complete revascularization. However, treating many lesions in a single session increases the risk of vascular injury. In fact, in this study, death occurred with the first session in one severe case. From a safety standpoint, it is reasonable to refrain from treating many vessels in a single session in severe cases. However, this increases the number of sessions required to achieve complete revascularization. The BPA revascularization strategies need to be further investigated.

### Limitations

There are some limitations to this study. First, this was a two-center study of patients with relatively moderate PH. Therefore, larger studies that include more patients with more severe CTEPH are needed. Second, a control population that did not undergo additional BPA after PH relief was not included, although previous reports have shown that hemodynamics after BPA therapy remain unchanged in the distant phase [10, 17]. Third, because the definition of the PH relief phase includes the use of vasodilators, variation in the PH relief phase status occurred. However, in clinical practice, treatment of many PH relief phase patients has been terminated, with a combination of incomplete BPA therapy and pulmonary vasodilators. This study may have demonstrated the significance of additional BPA from the PH relief phase. Fourth, the effect on residual lesions involves a combination of re-dilation of insufficiently dilated sites and expansion of untreated sites; this study did not examine the relative contributions of these processes. Fifth, the end-point of BPA therapy in this study was subjective. The treatment of some patients was terminated when type D lesions were not treated.

## Conclusions

In patients with CTEPH, complete revascularization with BPA beyond PH relief is beneficial because it may improve symptoms, hemodynamics, oxygenation, and exercise capacity and reduce the need for pulmonary vasodilators and oxygen therapy.

## Supporting information

S1 Dataset (XLSX)
